# Matrix stiffness regulates arteriovenous differentiation of endothelial progenitor cells during vasculogenesis in nude mice

**DOI:** 10.1111/cpr.12557

**Published:** 2018-11-28

**Authors:** Changyue Xue, Qian Huang, Tao Zhang, Dan Zhao, Quanquan Ma, Taoran Tian, Xiaoxiao Cai

**Affiliations:** ^1^ State Key Laboratory of Oral Diseases, National Clinical Research Center for Oral Diseases, West China Hospital of Stomatology Sichuan University Chengdu China; ^2^ Jiangsu Key Laboratory of Oral Diseases Nanjing Medical University Nanjing China; ^3^ Department of Implantology, Affiliated Hospital of Stomatology Nanjing Medical University Nanjing China

**Keywords:** arteriovenous development, matrix characteristics, Ras/Mek pathway, tissue engineering, vasculogenesis

## Abstract

**Objectives:**

The aim of the study was to investigate the effect of matrix stiffness on arteriovenous differentiation of endothelial progenitor cells (EPCs) during vasculogenesis in nude mice.

**Materials and methods:**

Dextran hydrogels of differing stiffnesses were first prepared by controlling the crosslinking reaction to generate different thioether bonds. Hydrogels with stiffnesses matching those of the arterial extracellular matrix and venous extracellular matrix were separately combined with mouse bone marrow‐derived EPCs and subcutaneously implanted on either side of the backs of nude mice. After 14 days, artery‐specific marker Efnb2 and vein‐specific marker Ephb4 in the neovasculature were detected to determine the effect of matrix stiffness on the arteriovenous differentiation of EPCs in vivo.

**Results:**

Fourteen days after the implantation of the EPC‐loaded dextran hydrogels, new blood vessels were observed in both types of hydrogels. We further verified that matrix stiffness regulated the arteriovenous differentiation of EPCs during vasculogenesis via the Ras/Mek pathway.

**Conclusions:**

Matrix stiffness regulates the arteriovenous differentiation of EPCs during vasculogenesis in nude mice through the Ras/Mek pathway.

## 1 INTRODUCTION

The establishment of functional vascularization is key for tissue regeneration and represents one of the major challenges to the broad implementation of tissue engineering in clinical practice.^1^ The formation of organ‐specific vasculatures requires crosstalk between the developing tissue and specialized endothelial cells (ECs). We propose a new source of specialized ECs based on bone marrow‐derived endothelial progenitors (EPCs). EPCs circulate in the bloodstream, proliferate and differentiate into mature ECs.^2^ After migrating into the peripheral circulation, EPCs assemble at sites of endothelial injury in response to stimuli for revascularization and endothelial repair. Previous studies have indicated that EPCs participate in the pathogenesis of vascular diseases, such as atherosclerosis, abdominal aortic aneurysm and cardiovascular diseases.^3^ EPCs may be divided into two populations as follows: early and late EPCs. Early EPCs, which can be obtained by culturing isolated mononuclear cells for 4‐7 days, have limited proliferative capacity. In contrast, late EPCs exhibit higher proliferative potential in culture and survive beyond 2 weeks. Both cell types contribute to neovasculogenesis in vivo; early EPCs secrete various angiogenic cytokines, and late EPCs differentiate into specific ECs.^4^ The phenotype of a late EPC is highly dependent on its microenvironment. However, the functional properties of EPCs and the molecular mechanisms of their specialized differentiation into arterial and venous subtypes are still unknown. This limitation negatively affects the practical use of EPCs. Previous studies have indicated that fluid shear stress induces the differentiation of EPCs into arterial ECs^5^; this finding suggests that controlling the physical characteristics of the microenvironment may represent a method for optimizing EPC‐based vascularization outcomes.

The vasculature is subdivided into two interconnected, yet structurally and functionally distinct, networks of arteries and veins. They form one of the body's largest surfaces, which serves as a critical interface between the circulation and the different organ microenvironments. Among the microenvironmental cues, matrix stiffness is a universal mechanical input that profoundly controls cell behaviour, including differentiation.^6^, ^7^ The range of venous tissue stiffness in mammals, which is between the stiffness of the epithelium and cartilage, ranges from 3 to 50 kPa.^8^, ^9^, ^10^ Unlike veins, arteries can withstand a higher blood pressure and are surrounded by several layers of smooth muscle cells and connective tissues.^11^ Therefore, arteries are stiffer, with stiffness ranging from approximately 50 to 150 kPa or higher.^12^, ^13^ However, when these stiffness values are compared with those of the glass or plastic containers used for tissue culture, such as Petri dishes (~10^6^ kPa), it becomes apparent that the microenvironment in which most cells are cultured in vitro is not representative of the physiological scenario. Accordingly, in order to design matrices to the specific requirements of diverse tissues, it is necessary to develop well‐defined biomaterials that mimic the matrices of the vasculature and enable the reliable control of functional vascularization.

With this in mind, we first developed a stiffness‐adjustable dextran hydrogel model. Mouse bone marrow‐derived EPCs were cultured in the hydrogels to form vascular networks following in vivo implantation to investigate the effect of matrix stiffness on the arterial‐venous differentiation of EPCs. We further explored the precise mechanism by which matrix stiffness regulates the expression of arterial and venous markers in the new vessels. Our work provides a potential method for adapting EPC‐based vascularization to the specific requirements of a diverse range of tissues, thus, representing a substantial advancement in regenerative medicine.

## 2 MATERIALS AND METHODS

### 2.1 Cell culture and identification

This investigation conformed to the governing ethical principles, and the protocols were approved by the Institutional Review Board (IRB) of Sichuan University.

Endothelial progenitor cells were obtained from 4‐week‐old female BALB/c mice provided by the Centre of Genetically Engineered Mice, Sichuan University, Chengdu, China. After cervical dislocation under anaesthesia (10% chloral hydrate, 3 mL/kg ip), the femurs of mice were flushed with PBS containing 1% bovine serum albumin (BSA) (Equitech‐Bio, Kerrville, TX, USA) and 1% penicillin/streptomycin (Hyclone, Logan, UT, USA) and were then filtered through sterile 200‐μm nylon mesh. The cell suspension was added to Ficoll (Sigma‐Aldrich, St. Louis, MO, USA) at a 1:1 (v/v) ratio to generate density gradients. The mixture was then centrifuged at 400 *g* for 20 minutes to separate the cells into three layers. Cells in the middle layer were extracted and cultured at an initial concentration of 1×10^6^ cells/mL in M199 medium (Hyclone) containing 10 ng/mL vascular endothelial growth factor 164 (VEGF 164, 493‐MV/CF, R&D Systems, Minneapolis, MN, USA), 3 ng/mL basic fibroblast growth factor (bFGF, 3139‐FB/CF, R&D Systems), 3 ng/mL insulin‐like growth factor (IGF, 791‐MG, R&D Systems), 10% foetal bovine serum (FBS) and 1% penicillin/streptomycin. All cultures were maintained in a standard humidified incubator at 37°C and 5% CO_2_.

For EPC characterization, cells were incubated with 1,1‐dioctadecyl‐3,3,3,3‐tetramethylindo‐carbocyanin‐eperchlorate‐labelled acetylated low‐density lipoprotein (Dil‐acLDL; 20 μg/mL; Invitrogen, Carlsbad, CA, USA) for 1 hour and fixed by 4% cold paraformaldehyde for 20 minutes. Cells were then washed with PBS and incubated with FITC‐labelled Ulex europaeus agglutinin‐1 (UEA‐1, 15 μg/mL; Sigma‐Aldrich) for another 1 hour. Cells were then washed and counterstained with 10 μg/mL Hoechst 33258 (Sigma‐Aldrich) for 10 minutes. Samples were visualized using a fluorescence microscope (Olympus, Tokyo, Japan). Fluorescent staining of CD34, a stem/progenitor cell marker, was performed to further confirm the stem cell phenotype. Furthermore, the expressions of CD31 and VE‐cad were analysed by flow cytometry. Briefly, cells were digested with trypsin to make cell suspension (2 × 10^7/^mL) and incubated with conjugated antibodies, including PE‐conjugated anti‐mouse CD31 (FAB3628P, R&D Systems) and APC‐conjugated anti‐mouse VE‐cad (FAB1002A, R&D Systems) for 30 minutes. For each antibody, a relevant isotype control was used. Finally, the cells were washed with PBS and analysed by BD FACSAria II (BD Biosciences, San Diego, CA, USA).

### 2.2 Fabrication of dextran hydrogels of varying stiffnesses

The hydrogel preparation method was based on the non‐cytotoxic crosslinking of maleimide‐modified dextran polymers with thiol‐reactive groups (mal‐dextran) and crosslinkers with thiol groups (CD‐link) (3‐D Life Hydrogel, pH 7.2, Reutlingen, Germany). To adjust the stiffness of the hydrogel‐based extracellular matrix (ECM), dextran hydrogel (300 μL) containing different concentrations of mal‐dextran and CD‐link was added. The gels were prepared by sequentially mixing sterile components (Table 1) such as (a) water, 10× concentration buffer, and the polymer of dextran were combined in a reaction tube and mixed well; (b) the cell suspension or medium was added; (c) CD‐link was added and the suspension was mixed by pipetting up and down; (d) once the gel formed, it was covered in cell culture medium; (e) the culture dish was placed in the incubator; (6) the medium was renewed after 50 minutes.

**Table 1:** Reagent volumes for the setup of hydrogels at different stiffness

Concentration of thioether (mmol/L)	1.0	2.0	3.0	4.0	5.0	6.0	7.0
Reagent		Volumes in μL					
Water	200	175	150	125	100	75	50
10 × Concentration buffer	25	25	25	25	25	25	25
Dextran (30 mmol/L)	10	20	30	40	50	60	70
Medium or cell suspension	50	50	50	50	50	50	50
CD‐link (20 mmol/L)	15	30	45	60	75	90	105
Total	300	300	300	300	300	300	300

The stiffness of the final mixture was estimated based on the weight‐induced compression of the matrix and calculated according to the equation:

$$E={\text{FL}}_{0}/A_{0}\Delta L,$$

where *E* is Young's modulus, *F* is the force exerted on the gel, *A*_0_ is the original cross‐sectional area through which the force is applied, Δ*L *is the change in the thickness of the gel and L_0_ is the initial thickness of the gel. The thicknesses of the hydrogel before and after force loading were determined microscopically.

### 2.3 Animal model

First, EPC‐containing dextran hydrogels were prepared. Based on the required ratios for the two groups of hydrogels at concentrations of 2.0 and 7.0 mmol/L (Table 1), we added water, 10 × concentration buffer, and mal‐dextran to a centrifuge tube and added the resulting mixture to a suspension of passage 1 EPCs (1 × 10^6^/mL) in M199 medium (Hyclone) supplemented with VEGF 164, bFGF and IGF. The CD‐link was then added and mixed while avoiding the formation of bubbles.

The 4‐week‐old female BALB/c nude mice were anaesthetized by injecting 5% chloral hydrate into the abdominal cavity. The left and right sides of the back skin were disinfected with 75% ethanol. Next, we subcutaneously injected the 300 μL hydrogel complexes in the left (6 kPa) and right (109 kPa) sides of the backs of the nude mice before crosslinking (Figure 3A). After 14 days, the hydrogels were surgically removed and washed with PBS.

### 2.4 Tissue sectioning

The hydrogels from the nude mice were fixed in 10% formaldehyde for 4 hour. After fixation, the hydrogels were dehydrated sequentially with 80%, 90%, 95% and 100% ethanol I, then 100% ethanol II and 100% ethanol III, and cleared with xylene I and xylene II for 30 minutes each. The samples were immersed in paraffin wax I for 1 hour and paraffin wax II for 5 hour. The samples were embedded in paraffin wax with the material surface for sampling facing downward. After the wax block cooled and solidified, the samples were frozen at −20°C and sliced to a thickness of 4 μm.

### 2.5 Immunohistochemical staining to identify in vivo vasculogenesis

The sections were dewaxed with xylene I for 20 minutes and xylene II for 20 minutes, then rehydrated with 100% ethanol I for 5 minutes, 100% ethanol II for 5 minutes, 95% ethanol for 5 minutes and 80% ethanol for 5 minutes, followed by washing with PBS. Endogenous peroxidase was blocked and inactivated by incubating the samples in 3% H_2_O_2_ at 37°C for 15 minutes. For antigen retrieval, the samples were boiled for 10 minutes in 0.01 mol/L citric acid buffer (pH 6.0). The prepared primary anti‐CD31 antibody (77699s, CST, USA) was added dropwise to the samples, which were incubated at 4°C overnight. The resulting mixture was allowed to settle for 30 minutes, washed with PBS, incubated with the secondary antibody at 37°C for 30 minutes. Next, the samples were stained with 3,3'‐diaminobenzidine (ZSGB‐BIO, Beijing, China); the progress of the reaction was monitored under a microscope until completion, and then, the samples were thoroughly rinsed with ultrapure water. Finally, the samples were counterstained with haematoxylin (Sigma, USA), dried, sealed and photographed.

### 2.6 Detection of arteriovenous markers with double‐label immunofluorescence staining

Sectioning was performed as described above. Then, the samples were dewaxed with xylene I for 20 minutes and xylene II for 20 minutes, then rehydrated with 100% ethanol I for 5 minutes, 100% ethanol II for 5 minutes, 95% ethanol for 5 minutes and 80% ethanol for 5 minutes, and washed three times with PBS. Endogenous peroxidase was blocked and inactivated by incubation with 3% H_2_O_2_ at 37°C for 15 minutes, and then, the samples were washed with PBS. For antigen retrieval, the samples were boiled in a 0.01 mol/L citrate buffer (pH 9.0) for 10 minutes. Sufficient volumes of diluted primary anti‐Efnb2 (rabbit monoclonal, ab150411, Abcam, Cambridge, MA, USA) and anti‐Ephb4 (rat monoclonal, ab106130) antibodies were added to the samples prior to simultaneous incubation overnight at 4°C. After incubation, the primary antibodies were removed, and the samples were washed with PBS. The samples were incubated with Alexa Fluor® 594 anti‐rabbit IgG and Alexa Fluor® 488 anti‐rat IgG (Invitrogen, 1:500 in PBS) for 1 hour. Starting with the addition of the fluorescently labelled secondary antibodies, all operating steps were performed in the dark. The specimens were incubated with Hoechst 33258 (10 μg/mL) for 15 minutes, then rinsed with PBS. The samples were imaged using confocal laser scanning microscopy (Leica TCS SP8, Wetzlar, Germany). The fluorescent staining was statistically analysed with ImageJ software.

### 2.7 Quantitative real‐time PCR

Briefly, total RNA was extracted from EPCs using the RNeasy Plus Mini Kit (Qiagen, Hilden, Germany), and the reverse transcription reaction was performed with the synthesis kit (Bio‐Rad, Hercules, CA, USA), as per the manufacturer's instructions. Quantitative PCR (qPCR) was performed with the Prime Script RTPCR Kit (TaKaRa, Tokyo, Japan) using the ABI 7300 PCR machine (Applied Biosystems, Foster City, CA, USA). Amplification of each target mRNA was performed according to the following steps: denaturation for 30 seconds at 95°C, followed by 42 cycles, consisting of 5 seconds at 94°C and 32 seconds at 60°C. For each reaction, a melting curve was generated to test for primer dimer formation and false priming. The sequences of the forward and reverse primers are listed in Table 2.

**Table 2:** Primer sequences of β‐actin and target genes

Target genes (mouse)	Primer pairs (5' → 3')
*β‐actin* (154 bp)	Forward: GGCTGTATTCCCCTCCATCG Reverse: CCAGTTGGTAACAATGCCATGT
*Efnb2* (118 bp)	Forward: ATTATTTGCCCCAAAGTGGACTC Reverse: GCAGCGGGGTATTCTCCTTC
*Ephb4* (106 bp)	Forward: CACAGCGACTTGGCTGCTA Reverse: AGGTGGGATCAGAGGAGTTCT
*Ras* (116 bp)	Forward: CAAGAGCGCCTTGACGATACA Reverse: CCAAGAGACAGGTTTCTCCATC
*Mek* (149 bp)	Forward: AAGGTGGGGGAACTGAAGGAT Reverse: CGGATTGCGGGTTTGATCTC
*RhoA* (138 bp)	Forward: AGCTTGTGGTAAGACATGCTTG Reverse: GTGTCCCATAAAGCCAACTCTAC
*Notch1* (74 bp)	Forward: GATGGCCTCAATGGGTACAAG Reverse: TCGTTGTTGTTGATGTCACAGT
*Hey1* (231 bp)	Forward: GCGCGGACGAGAATGGAAA Reverse: TCAGGTGATCCACAGTCATCTG
*VEGFR3* (182 bp)	Forward: CTGGCAAATGGTTACTCCATGA Reverse: ACAACCCGTGTGTCTTCACTG

### 2.8 Western blot analysis

Protein samples were mixed with Bio‐Rad Laemmli Sample Buffer and then boiled at 100°C for 5 minutes. Proteins were separated by electrophoresis in 10% acrylamide gels containing SDS and transferred onto polyvinylidene difluoride membranes at 200 mA for 1 hour. Membranes were blocked with 5% BSA for 1 hour prior to incubation with 1:500‐1000 antibodies (Abcam) including Efnb2 (ab150411), Ephb4 (ab106130), Ras (ab16907), MAP kinase‐ERK kinase (Mek, ab178876), Ras homologue family member A (RhoA, ab187027), Notch1 (ab52627), hairy/enhancer‐of‐split related with YRPW motif 1 (Hey1, ab154077), vascular endothelial growth factor receptor 3 (VEGFR3, ab91124) and glyceraldehyde‐3‐phosphate dehydrogenase (GAPDH, ab181602) for 3 hour. The membranes were then washed with TBST and probed with appropriate secondary antibodies for 1 hour. The blots were developed using the Western Blotting Luminol Reagent Kit (Santa Cruz Biotechnology, Santa Cruz, CA, USA). Signals were visualized using Kodak X‐AR, and signal intensity was analysed with the Quantity One 4.6.3 software (Bio‐Rad). The abundance of each protein of interest was compared to that of the loading control (GAPDH) based on the relative intensities of the bands.

### 2.9 Artificial degradation of dextran hydrogels

The hydrogels were artificially degraded with dextranase (3‐D Life Hydrogel, Reutlingen, Germany) to extract mRNA and protein. First, the dextranase was diluted in M199 medium (v/v, 1:20). Then, the hydrogels were soaked in the dextranase solution (3 mL; v/v, 1:10) at 37°C for 50 minutes. After the hydrogels had dissolved, we subjected the resulting tissues to centrifugation and washed twice with PBS to ensure effective removal of the dextranase and the dissolved gel components.

### 2.10 Inhibitor experiments

We treated the animal experimental group implanted with 109 kPa hydrogel with FTS (20 μmol/L, diluted with PBS) and the control group implanted with 6 kPa hydrogel with PBS. Starting on day 2, we injected the hydrogel samples implanted in the nude mice with the FTS solution (right) and PBS (left) every 24 hour (injection volume, 150 μL/side). After 14 days, the hydrogels were removed and digested as above. The samples were subjected to quantitative polymerase chain reaction (qPCR) and Western blotting to detect the gene transcription and protein expression levels of Efnb2 and Ephb4.

### 2.11 Statistical analysis

All experiments were repeated for at least three times, independently. Statistical analysis of data was performed with the SPSS statistical software version 21.0 (IBM, Armonk, NY, USA) using t test. In each analysis, data were considered to be significantly different when the two‐tailed *P* value was <0.05.

## 3 RESULTS

### 3.1 Identification of EPCs

**Figure 1:**
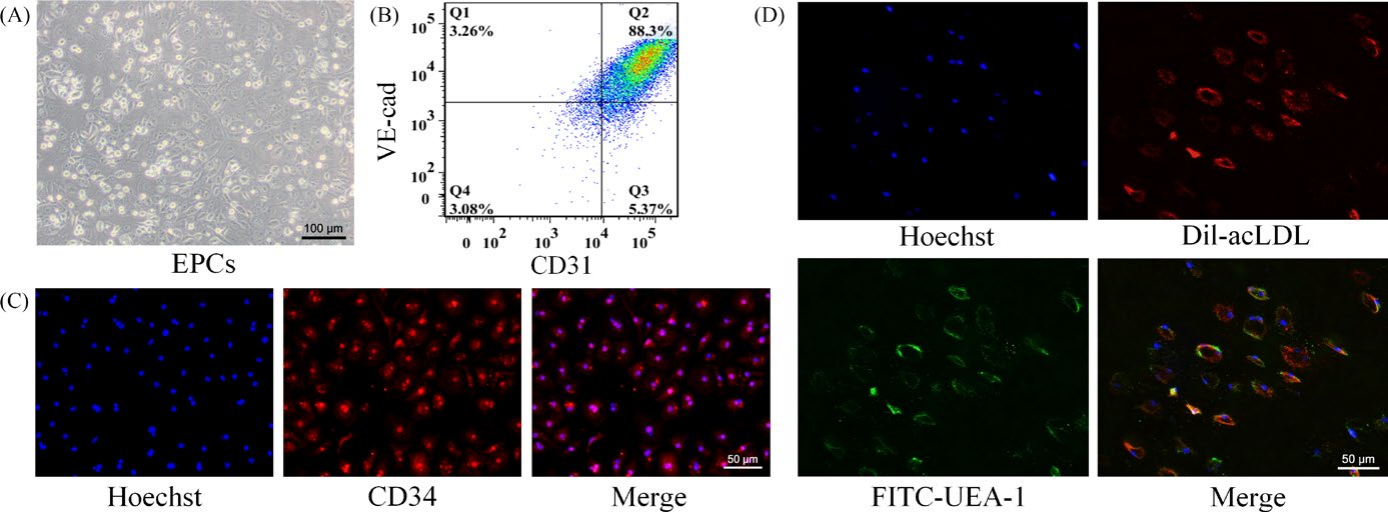
Characterization of EPCs. A, Image of the EPCs observed under an inverted light microscope; B, Flow cytometry for CD31 and VE‐cad expression of EPCs. Percentage of CD31^+^/VE‐cad^+^ cells among total EPCs is indicated (Q2); C, Positive staining for the stem/progenitor cell marker CD34; D, Ulex europaeus agglutinin‐1 binding (green) and 1,1‐dioctadecyl‐3,3,3,3‐tetramethylindo‐carbocyanin‐eperchlorate‐labelled acetylated low‐density lipoprotein uptake (red) in EPCs

Image of the obtained cells observed by a microscope was shown in Figure 1A. Flow cytometric analysis first revealed that CD31^+^/VE‐cad^+^ double positive cells accounted for 88.3% of the total population, demonstrating that the cultured cells possessed endothelial cell characteristics (Figure 1B). The attached cells also stained positive for the stem/progenitor cell marker CD34 (Figure 1C). The bone marrow‐derived EPCs were further characterized by Dil‐acLDL uptake as well as UEA‐1 binding. The double staining results confirmed that the attached cells displayed typical phenotypic and functional properties of EPCs.

### 3.2 Assessment of the dextran hydrogels of tunable stiffness

**Figure 2:**
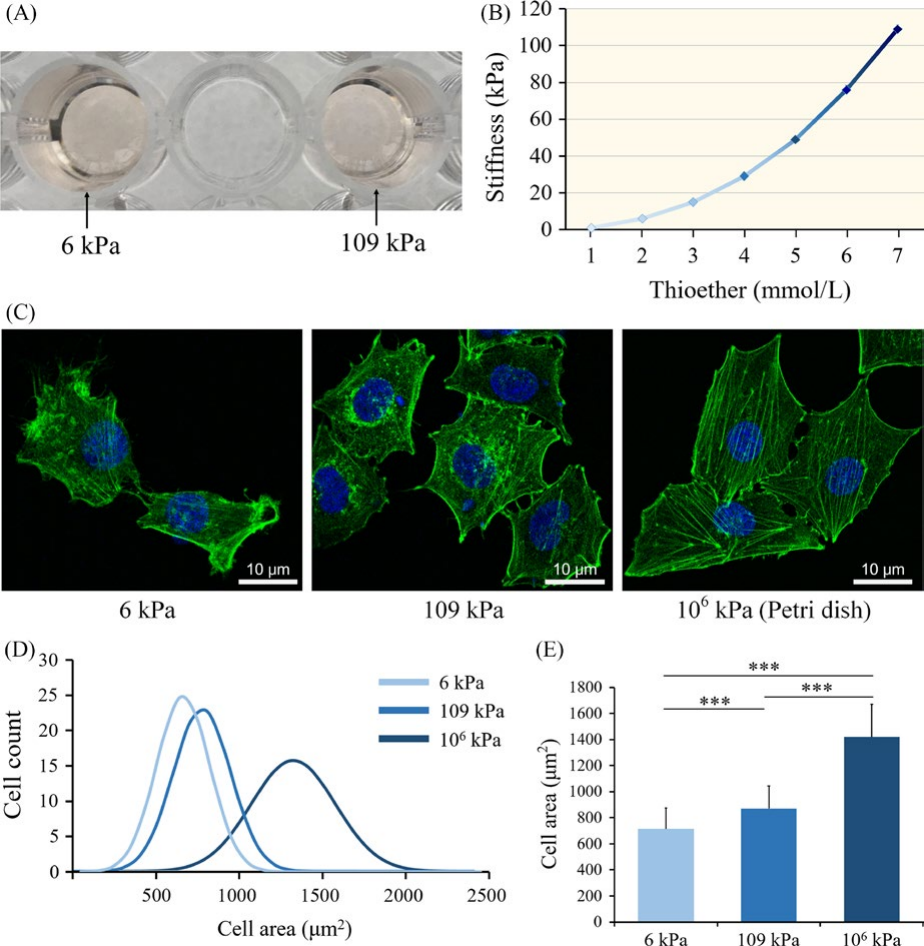
A, Two dextran hydrogels with different stiffnesses after crosslinking; B, Stiffnesses of dextran hydrogels with different dextran (thioether bond) contents; five different samples were tested as indicated; C, Fluorescent images of the cytoskeleton (phalloidin staining of F‐actin) of EPCs on matrix of the indicated stiffness. Scale bars are 20 μm; D, Distributions of cell area of EPCs on matrices of varying degrees of stiffness; E, Quantitative assessment of cell morphology; 100 cells were measured in each group. *P < 0.05, **P < 0.01, ***P < 0.001. Data shown are representative of three independent experiments

The hydrogels with different compositional ratios were added to a 48‐well plate. After 50 minutes, the hydrogels were fully crosslinked. Figure 2A shows two fully crosslinked dextran hydrogels (concentrations of 2.0 mmol/L and 7.0 mmol/L), which were both transparent and light pink in colour. The hydrogels with different dextran (thioether bond) contents of 1.0, 2.0, 3.0, 4.0, 5.0, 6.0 and 7.0 mmol/L had stiffnesses of 0.8 ± 0.2, 6.3 ± 0.7, 15.4 ± 0.8, 29.4 ± 0.9, 48.9 ± 1.1, 76.1 ± 2.4 and 108.6 ± 2.3 kPa, respectively, indicating an increase in hydrogel stiffness with higher dextran (thioether bond) contents (Figure 2B).

Moreover, the hydrogels with concentrations of 2.0 and 7.0 mmol/L had stiffnesses in the ranges of the venous ECM and arterial ECM, respectively. Therefore, in the following in vivo experiments, we selected these two dextran hydrogels to mimic the venous and arterial ECM for EPC cultures, which we referred to as the 6 kPa group and 109 kPa group, respectively.

### 3.3 Analysis of EPC morphology on matrices of varying stiffness

First, we investigated the response of EPCs to microenvironmental stiffness via the dynamics of the cytoskeletal network. Phalloidin staining of the cell cytoskeleton indicated that there were apparent differences in cellular morphology and cytoskeletal filament arrangements on matrices of varying stiffness. The EPCs cultured on the surfaces of the soft matrices were ovoid with a small cell spreading area, and their internal cytoskeletal filaments were short, slender, and were unable to form obvious filament bundles. On stiffer matrices, the cytoskeleton filaments were elongated and formed crosslinked filament bundles and a larger EPC spreading area (Figure 2C). In addition, we quantitatively analysed the cell spreading area and reached the same conclusion: cell spreading area increases with increasing matrix stiffness (Figure 2D‐E).

### 3.4 In vivo vasculogenesis during culture of the hydrogel‐EPC composites

**Figure 3:**
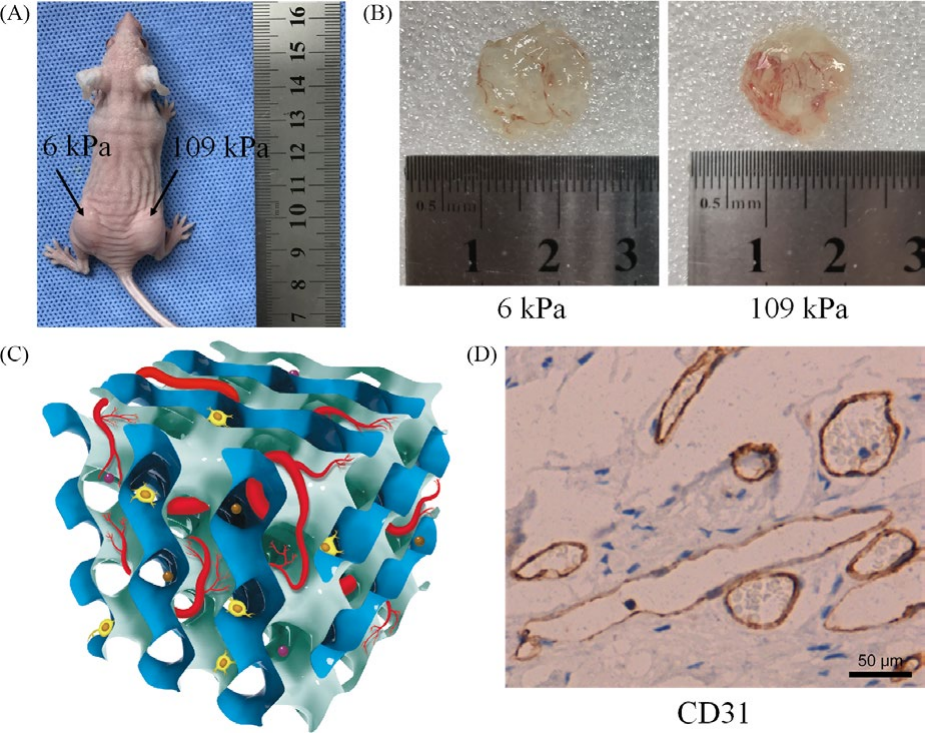
A, Injection of the hydrogel‐EPC composites on either side of the backs of nude mice; B, Vasculogenesis status after 14 d of hydrogel implantation; C, Schematic representation of the vasculogenesis model in the hydrogel support material. Brown and purple balls represent vascular growth factors; D, Immunohistochemical staining of CD31 in hydrogel (6 kPa) slices. Scale bar is 50 μm

The hydrogels with different stiffnesses were translucent, and blood vessels were visible in both the soft and hard hydrogels (Figure 3B‐C). Next, we assessed the nature of the tubular structures within the hydrogels by immunohistochemistry. The tubular structures in the hydrogels were labelled with the anti‐CD31 antibody (Figure 3D), demonstrating that the newly formed tubular structures in the hydrogels were vascular structures.

### 3.5 Detection of the arteriovenous markers Efnb2 and Ephb4

**Figure 4:**
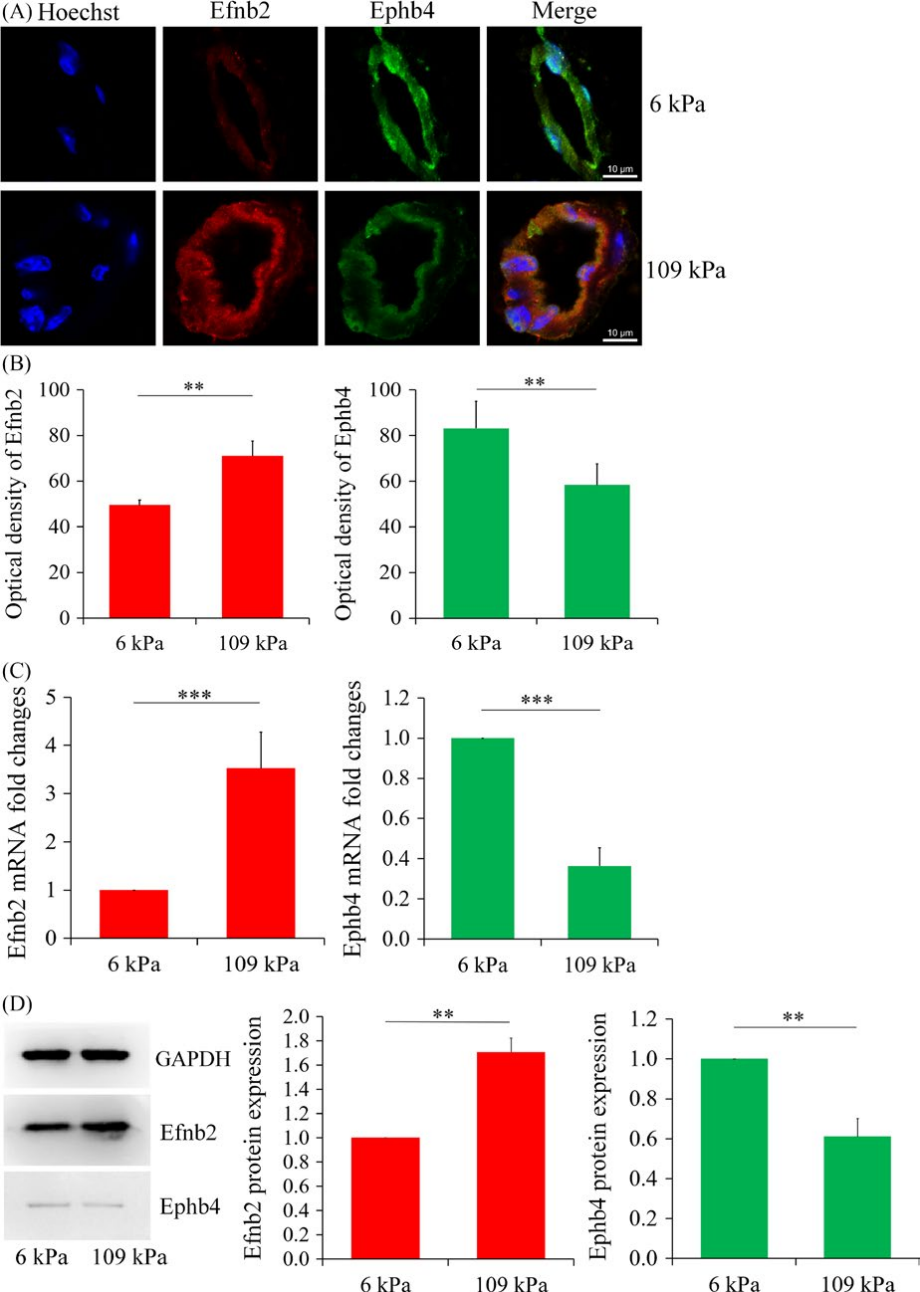
A, Double immunofluorescence staining of arteriovenous marker proteins (scale, 10 μm) in hydrogels with different stiffnesses; B, Statistical analysis of the fluorescence intensities. Each experimental value is expressed as the mean ± standard deviation; C, Gene transcript levels of the arteriovenous markers in the newly formed vessels in the hydrogels of different stiffnesses. All groups of genes were first normalized to internal references, then normalized to the control group (6 kPa group); D, Western blotting for and statistical analysis of Efnb2 and Ephb4 expression in the newly formed vessels in the hydrogels of different stiffnesses. Data shown were representative of three independent experiments; *P < 0.05, ** *P* < 0.01, ***P < 0.001

In our study, new vessels cultured on both hydrogels simultaneously expressed the arterial endothelial marker Efnb2 and the venous endothelial marker Ephb4. The expression of Efnb2 in the 109 kPa hydrogel was higher than that in the 6 kPa hydrogel, whereas Ephb4 showed the opposite expression pattern (Figure 4A‐B).

Real‐time qPCR analysis indicated that the mRNA level of Efnb2 in the 109 kPa group was 3.63 × higher than that in the 6 kPa group. In contrast, the mRNA level of Ephb4 in the 109 kPa group was 36.4% of that in the 6 kPa group. Thus, the mRNA expression of the Efnb2 gene increased with the stiffness of the hydrogel matrix, whereas the transcription level of the Ephb4 gene showed the opposite trend (Figure 4C).

Western blotting further confirmed that the expression of the arterial marker increased with increasing matrix stiffness, whereas the venous marker showed the opposite trend. Consistent with the results of immunofluorescence staining, the protein expression levels of Efnb2 and Ephb4 in the 109 kPa group were 1.71 × and 0.61 × those in the 6 kPa group, respectively (Figure 4D).

### 3.6 Mechanotransduction and underlying intracellular signalling pathways

**Figure 5:**
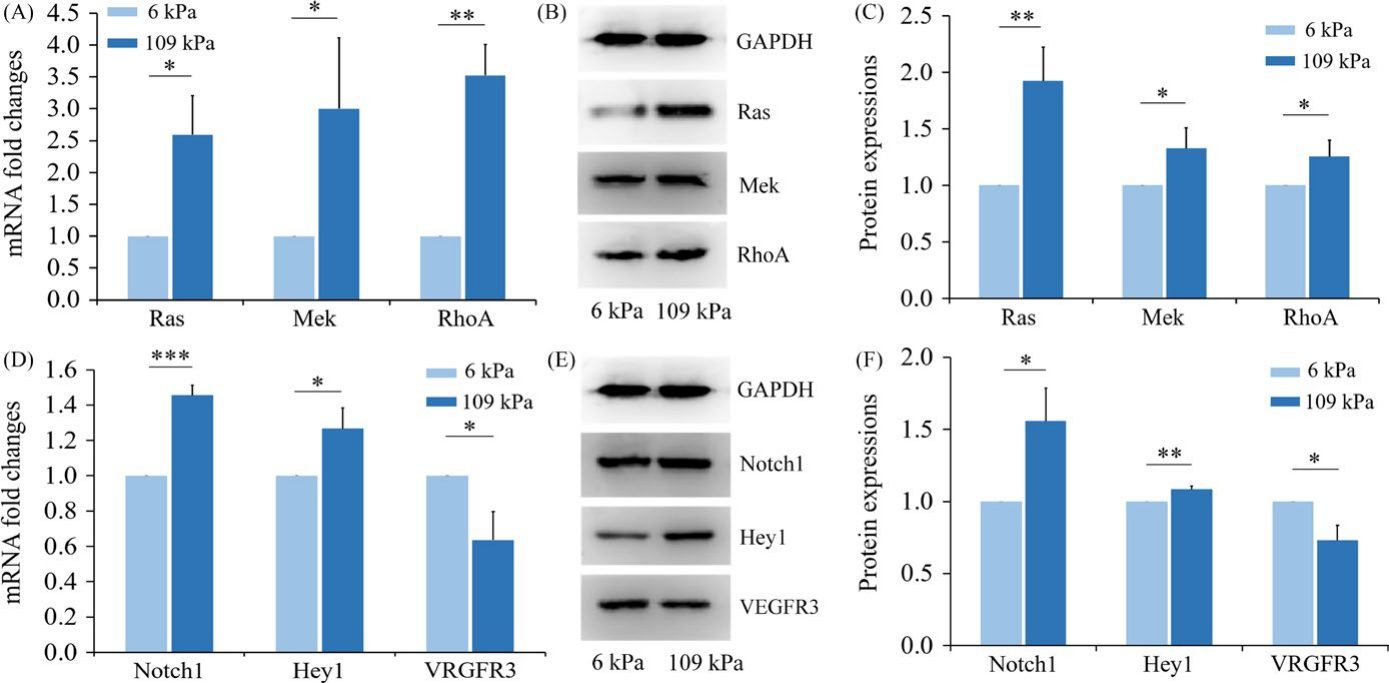
Small GTP‐binding protein pathways and Notch signal transduction are activated by matrix with high stiffness. A, Real‐time PCR analyses of Ras, MAP kinase‐ERK kinase (Mek) and Ras homologue family member A (RhoA) expression; B, Western blot analyses showing protein expression of Ras, Mek and RhoA; C, The protein expressions of Ras, Mek and RhoA were quantitated, and data are shown as a histogram. Each experimental value is expressed as the mean ± standard deviation; D, Real‐time PCR analyses of Notch1, hairy/enhancer‐of‐split related with YRPW motif 1(Hey1) and vascular endothelial growth factor receptor 3 (VEGFR3) expression; E, Western blotting of Notch1, Hey1 and VEGFR3; F, Protein expressions of Notch1, Hey1 and VEGFR3 were quantitated and data are shown as a histogram. *P < 0.05, **P < 0.01, ***P < 0.001. Data shown are representative of three independent experiments

Next, we investigated the potential signalling pathways involved in the mechanotransduction process. In our study, the expressions of Ras and Mek were upregulated in the 109 kPa group at both the mRNA and protein levels (Figure 5A‐C). The upregulation of RhoA could also be observed, which further contributed to the activation of the Ras pathway.^14^, ^15^ A number of other studies have confirmed that changes in the Ras/Mek pathway affect the Notch pathway and that activation of the Notch pathway promotes arterial differentiation and inhibits the expression of vein‐related marker proteins.^16^ In this work, we found that both the mRNA and the protein levels of Notch1 were elevated in the 109 kPa group along with its downstream protein Hey1. Further, consistent with our previous study and other experimental reports,^17^, ^18^, ^19^, ^20^ activation of the Notch pathway can inhibit the expression of VEGFR3 (Figure 5D‐F).

### 3.7 Results of inhibitor experiments

**Figure 6:**
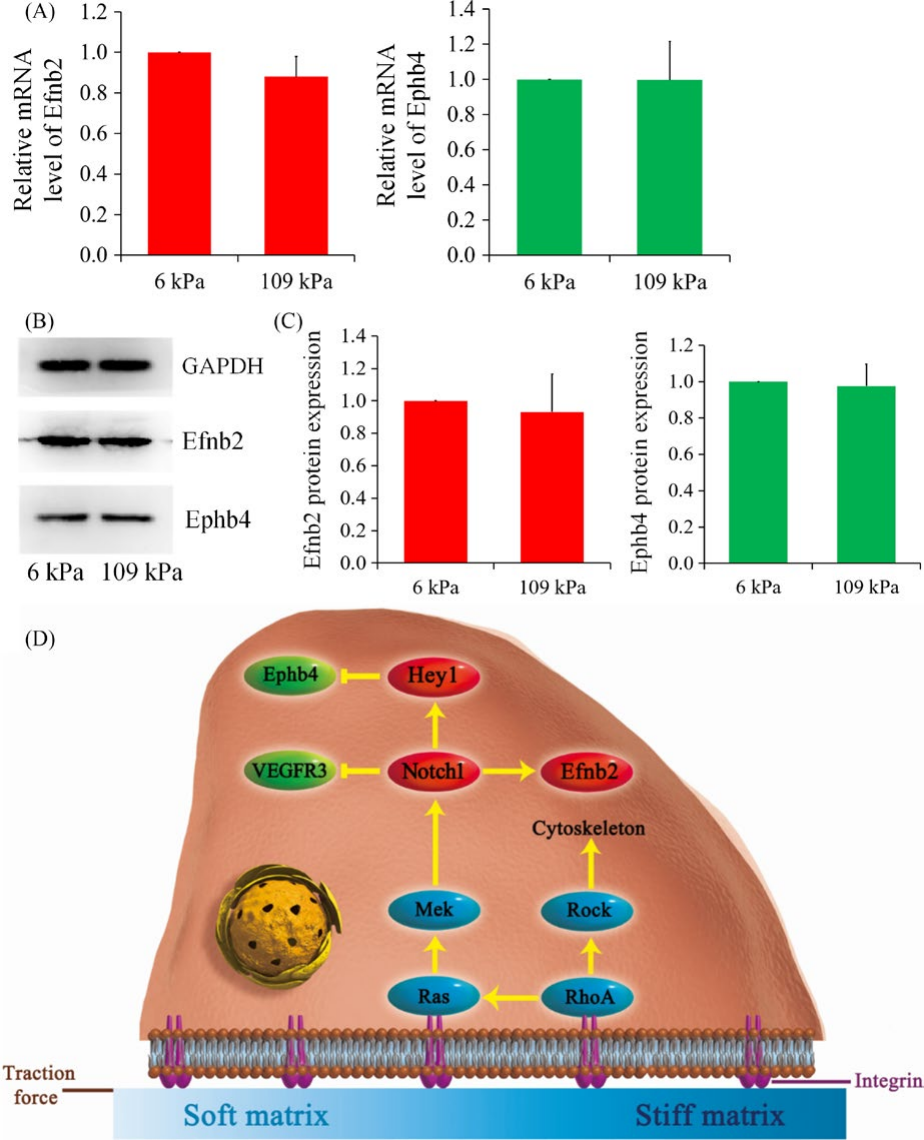
Results of inhibitor study. A, The mRNA levels of Efnb2 and Ephb4 both showed no difference between 6 kPa group and 109 kPa group different after treatment with farnesylthiosalicylic acid; B, C, Western blot analyses also showed that the regulatory role of matrix stiffness was blocked after treatment with farnesylthiosalicylic acid. Data shown are representative of three independent experiments; D, Schematic diagram illustrating the mechanism by which the arteriovenous differentiation of EPCs during vasculogenesis is regulated in response to matrix stiffness

The Ras signalling inhibitor farnesylthiosalicylic acid (FTS) was used to verify the role of the Ras/Mek signalling pathway in the matrix stiffness‐mediated regulation of the arteriovenous differentiation of EPCs. In the experimental 109 kPa group treated with the Ras/Mek pathway inhibitor FTS, the Efnb2 and Ephb4 gene levels did not statistically differ from those in the control 6 kPa group (Figure 6A). Western blotting revealed that treatment with the inhibitor prevented significant enhancement of the expression of the arterial marker Efnb2 in the hard hydrogel and even slightly decreased its expression (Figure 6B). There were no significant differences in the expression of Efnb2 or Ephb4 between the two groups in the presence of the inhibitor. Treatment with the inhibitor FTS eliminated the effect of matrix stiffness on both arterial and venous phenotypes, demonstrating the important role of Ras/Mek in the matrix stiffness‐regulated arteriovenous differentiation of EPCs.

## 4 DISCUSSION

### 4.1 Dextran hydrogel properties

As a necessary microenvironment for cells, the ECM provides mechanical support for cells and conducts a variety of biochemical and biophysical signals, thereby affecting the biological behaviour of cells.^21^ In recent years, the interactions between the physical properties of the ECM and cells have received extensive attention. To date, in vitro research has typically utilized traditional methods in which cells are inoculated on plastic or glass Petri dishes or flasks with stiffnesses reaching magnitudes of gigapascals—much higher than the stiffness of the ECM of the tissues and organs in the body. The use of traditional glass or plastic as the matrix to explore cellular behaviour may introduce considerable bias compared to the true behaviour in normal physiological environments.

The 3‐D Life biomimetic hydrogel system used in this study is a matrix material that mimics ECM components to facilitate three‐dimensional cell cultures. Various stiffness levels can be obtained by varying the compositional ratio of dextran to its crosslinker in the hydrogels. A number of recent studies have used this type of hydrogel and confirmed that its high biocompatibility prevents the adverse effects of material toxicity on cells.^22^, ^23^, ^24^

### 4.2 Effect of matrix stiffness on cell morphology

The process through which cells respond to ECM stiffness is also the process through which cells achieve a dynamic balance between their contraction tension and the ECM force.^25^ When the stiffness of the material changes, mechanical signals are transduced to the cells via integrins that affect cytoskeletal assembly and spreading behaviour. Many studies have shown that the Rho family of GTPases, a collection of switch molecules for cellular mechanical signalling, directly transmits stiffness signals into cells and regulates cytoskeletal organization.^26^, ^27^ And it is widely accepted that the Rho/Rock signalling pathway plays an important role in the regulation of cytoskeletal rearrangements and cell–matrix force transmission. The upregulation of RhoA activity in cells in a hard matrix has been confirmed in this study. Microstructures, such as microfilaments, microtubules and intermediate filaments, in the cytoskeleton support many biological reactions and microstructural changes affect downstream signalling molecules and regulate the transcription of related genes. Many basic cellular physiological processes, such as proliferation, differentiation, migration and apoptosis, can be affected by changes in the cytoskeleton.^28^, ^29^

### 4.3 In vivo vasculogenesis during culture of the hydrogel‐EPC composites

Vasculogenesis is the process in which desired new blood vessels are produced by close interactions between ECM, seed cells and growth factors.^30^ We performed a number of preliminary experiments in this study to determine the optimal experimental conditions for in vivo vasculogenesis in nude mice. We found that the EPC‐free hydrogels implanted into nude mice did not show vascular structure formation after 14 days, even when the hydrogels were seeded with vascular growth factors, such as VEGF and bFGF, and only a small number of cells and loose tissues were observed under the microscope. In contrast, when mouse bone marrow‐derived EPCs were inoculated into a dextran hydrogel containing vascular growth factors, and implanted in nude mice, newly formed blood vessel structures were observed in both soft and hard hydrogels after 14 days of culture in vivo. Immunohistochemical staining confirmed the expression of CD31 in blood vessel‐like structures with cavities filled with red blood cells, indicating the formation of functional blood vessels in the hydrogels.

### 4.4 Regulatory effects of matrix stiffness on the expression of arteriovenous markers in new vessels

Studies have confirmed that, as early as the early stage of embryonic development, venous ECs can specifically express Ephb4, and arterial ECs can specifically express Efnb2. These two molecules are considered specific markers that distinguish between venous and arterial vessels.^31^ EPCs can simultaneously express Efnb2 and Ephb4. The intracellular expression of Efnb2 inhibits that of Ephb4 and vice versa.^32^, ^33^ In the early stage of the formation of the vascular circulatory system, the balance between Efnb2 and Ephb4 expression guides ECs in the axial or other spatial direction, thereby forming the genetic basis for arterial and venous differentiation.^34^

In this experiment, we examined the gene and protein levels of arteriovenous markers in the blood vessels that were generated in vivo. Efnb2 and Ephb4 had opposite expression patterns in dextran hydrogels of differing stiffnesses. The expression of Efnb2 increased and the expression of Ephb4 decreased with increased matrix stiffness, which indicates that a stiffer matrix can promote the differentiation of EPCs into arteries, whereas a softer matrix can promote differentiation into veins.

### 4.5 Mechanism exploration

Studies on the morphologies of EPCs on matrices of different stiffnesses have shed light on the important role of the ECM‐integrin‐actin system for the transduction of matrix stiffness mechanical signals.^35^ The interactions between EPCs and the ECM initiate a series of reactions to convert mechanical signals into biochemical signals that affect the outcomes of cells. External physical signals from the ECM activate the superfamily of small GTP‐binding proteins through integrins.^36^ The superfamily of small GTP‐binding proteins can be divided into three major subfamilies as follows: Ras, Rho and Rab.^37^ Ras molecules are members of the superfamily of small GTP‐binding proteins, and the related signal transduction pathway Ras/Raf/Mek/Erk, also known as the Ras/Mek signalling pathway, is considered the “master switch” of many mechanically transduced signals.^38^ Moreover, the Notch pathway, which is closely associated with the Ras/Mek pathway, is an important signalling pathway in the regulation of vasculogenesis, vascular growth and differentiation.^39^ The Notch1 protein is an arterial marker. Therefore, matrix stiffness as a physical signal may be linked to arterial and venous phenotypes through the Ras/Mek signalling pathway and the Notch pathway.^40^

Ras and RhoA can be directly activated by matrix stiffness signals and the upregulation of RhoA activates Ras, which means that matrix stiffness can activate the Ras signalling pathway directly and indirectly.^41^, ^42^, ^43^ A number of other studies have confirmed that changes in the Ras/Mek pathway affect the Notch pathway and that activation of the Notch pathway promotes arterial differentiation and inhibits the expression of vein‐related marker proteins.^16^

Based on our results and the published literature, we propose a mechanism for the regulation of arteriovenous differentiation of EPCs by matrix stiffness (Figure 6). First, EPCs are linked to the ECM via integrins and ECM stiffness, a physical signal, enters cells via integrins. This activates the mechanical signalling pathways of small GTP‐binding proteins, namely the Ras/Mek pathway and the RhoA/Rock signalling pathway, and the activation of RhoA/Rock signalling directly affects cytoskeletal arrangement and cell spreading area and indirectly activates the Ras/Mek signalling pathway.^44^, ^45^, ^46^, ^47^ When activated, the Ras/Mek mechanical transduction pathway continues to upregulate the intracellular expression of Notch pathway‐related molecules, such as Notch1 and Hey1, which are closely related to the vascular system. Previous studies have shown that the expression of Notch1 positively correlates with that of Efnb2 and is correlated with VEGFR3 in a negative feedback manner and that Hey1 expression negatively correlates with Ephb4 expression. Based on this evidence, we propose that arteriovenous differentiation of EPCs is regulated by matrix stiffness via the Ras/Mek signalling pathway.^48^, ^49^, ^50^, ^51^

Notch1 and Hey1 are arterial markers, and VEGFR3 is a venous marker. The trends we detected in the changes in the expression of these markers were largely consistent with the trends in the changes of Efnb2 and Ephb4 expression. Enhanced arterial marker expression and decreased venous marker expression are associated with increased matrix stiffness. Therefore, the results of our research on the mechanism of action support our conclusion that the arteriovenous differentiation of EPCs is regulated by matrix stiffness.

## 5 CONCLUSIONS

In summary, matrix stiffness regulates the arteriovenous differentiation of endothelial progenitor cells during vasculogenesis in nude mice through the Ras/Mek pathway: arterial lineages were obtained on stiff substrates while venous commitment predominated in the softer matrix. Therefore, the stiffness of the matrix must be considered when conducting vascularized material design, drug testing and cell therapies to meet tissue‐ and organ‐specific vascularization requirements.

## CONFLICT OF INTERESTS

The authors declare that they have no competing interests.
